# Novel *Acinetobacter parvus* HANDI 309 microbial biomass for the production of *N*-acetyl-β-d-glucosamine (GlcNAc) using swollen chitin substrate in submerged fermentation

**DOI:** 10.1186/s13068-017-0740-1

**Published:** 2017-03-09

**Authors:** Tae Il Kim, Kwang Seok Ki, Dong Hyun Lim, Mayakrishnan Vijayakumar, Seong Min Park, Sun Ho Choi, Ki Young Kim, Seok Ki Im, Beom Young Park

**Affiliations:** 1 Dairy Science Division, National Institute of Animal Science, Rural Development Administration, #114, Shinbang 1Gil, Seonghwan-eup, Seobuk-gu, Cheonan-si, Chungcheongnam-do 331-801 South Korea; 2 Grassland and Forage Division, National Institute of Animal Science, Rural Development Administration, #114, Shinbang 1Gil, Seonghwan-eup, Seobuk-gu, Cheonan-si, Chungcheongnam-do 331-801 South Korea

**Keywords:** Chitinase, *Acinetobacter parvus*, Chitin, *N*-acetyl-d-glucosamine, Feces, Calves

## Abstract

**Background:**

*N*-acetyl-β-d-glucosamine (GlcNAc)_6_ is extensively used as an important bio-agent and a functional food additive. The traditional chemical process for GlcNAc production has some problems such as high production cost, low yield, and acidic pollution. Therefore, to discover a novel chitinase that is suitable for bioconversion of chitin to GlcNAc would be of great value.

**Results:**

Here, we describe the complete isolation and functional characterization of a novel exo-chitinase from *Acinetobacter parvus* HANDI 309 for the conversion of chitin. The identified exo-chitinase mainly produced *N*-acetyl-d-glucosamine, using chitin as a substrate by submerged fermentation. The *A. parvus* HANDI 309 biofuels producing exo-chitinase were characterized by TLC, and was further validated and quantified by HPLC. Furthermore, the optimal temperature and pH for the exo-chitinase activity was obtained in the culture conditions of 30 °C and 7.0, respectively. The maximum growth of the stationary phase was reached in 24 h after incubation. These results suggest that *A. parvus* HANDI 309 biofuels producing exo-chitinases may have great potential in chitin to *N*-acetyl-d-glucosamine conversion.

**Conclusions:**

The excellent thermostability and hydrolytic properties may give the exo-chitinase great potential in chitin to GlcNAc conversion in industry. This is the first report that *A. parvus* HANDI 309 is a novel bacterial strain that has the ability to produce an enormous amount of exo-chitinase-producing bio-agents in a short time on an industrial scale without any pretreatment, as well as being potentially valuable in the food and pharmaceutical industries.

**Electronic supplementary material:**

The online version of this article (doi:10.1186/s13068-017-0740-1) contains supplementary material, which is available to authorized users.

## Background

Chitin is the secondmost abundant, readily obtained, renewable and high molecular weight natural polysaccharide, consisting of β-1,4-linked *N*-acetyl-d-glucosamine (GlcNAc) with high similarity to cellulose, except that the glucose residue with hydroxyl groups is replaced by an acetylated or deacetylated amino group. It is dispersed in various locations, including snails, crustaceans, insects, vertebrates, plants and microorganisms [1]. For example, shellfish contain 20–58% of chitin, while fungal cell walls consist of 22–40% of chitin [2, 3]. In recent years, numerous researchers have been reported the use of chitin and related materials in various fields, including heavy metal recovery, drug delivery, wound healing, dietary fibre, agriculture andthe environment [4]. The natural biopolymer of chitin is composed of biological molecules such as carbohydrates, lipids, proteins and pigments, as well as minerals insoluble in water, or in the majority of organic solvents, so its uptake is not easy. Therefore, chitin demineralization and deproteinization are a vital process to acquire purified chitin. Also, the purification of chitin accomplished by using acids or alkalis has safety and environmental problems [3]. Hence, low-cost chitin-degrading enzymes are alternatively used for the production of chitin on an industrial scale.

Chitinases are a group of enzymes (EC.3.2.14) that hydrolyze the chitin to low-molecular-weight oligo and monomeric components and have been shown to be produced by some microorganisms, fungi, insects, higher plants and animals in which they play a significant physiological role depending on their origin [5]. The extracellular chitinase-producing microbes will use chitin or colloidal chitin as a carbon sources for the production of a mixture of chitinases and *N*-acetylglucosaminidase. These extra cellular chitinases can be classified into two types: intra- and extracellular. Endochitinases play a significant role in the cleavage of chitin to generate multimers of GlcNAc. Extracellular chitinases are mainly involved in producing GlcNAc, chitobiose or chitotriose. Chitinases have huge potential industrial applications, e.g. in the preparation of pharmaceutically relevant chitooligosaccharides and GlcNAc, the preparation of single cell protein (SCP), in cell differentiation, and they are also involved in isolating the protoplasts from fungi and yeast, in chitinous waste treatment, and in the control of malaria transmission [2]. GlcNAc is one of the primary products of enzymatic hydrolysis of chitin.

In recent years, GlcNAc has received more attention, due to its valuable functions in a broad range of fields, including the pharmaceutical and biotechnological industries, and especially in the fermented food industry its use is growing rapidly [6]. Generally, GlcNAc has been commercially produced by the acid hydrolysis of chitin with high concentrations abd high temperatures. However, this development has some demerits, including high cost, low yield and environmental pollution. Moreover, GlcNAcs manufactured by chemical methods limit their applications in the food industry [7, 8]. Hence, the bacteria-produced extracellular chitinase enzymes for nutritional functions can consume chitin as a substrate for the carbon source. Chemically-based pesticides cause long-lasting side effects on ecosystems. Therefore, researchers have paid more attention to searching for and developing non-hazardous, eco-friendly options. Such eco-friendly alternatives are considered as biofuels for bio-control agents which will inhibit or kill pests in a biologically safe manner and without producing any environmental pollution [3]. Hence, the present study was performed to characterize GlcNAc-produced chitinolytic chitinase by a novel antibiotic resistance method using *Acinetobacter parvus* HANDI 309 from the feces of calves. We also optimized the production and growth conditions.

## Methods

### Preparation of swollen chitin

The preparation of swollen chitin followed the method of Monreal and Reese [9]. Briefly, 10 g of flake chitin was added into 100 mL of 85% phosphoric acid at 4 °C for 48 h. Then, the swollen chitin was precipitated by adding the gelatinous mixture into an excess of cold water. The excess of water was then removed and the precipitate was washed with distilled water until the pH became neutral.

### Microorganism and production conditions of chitinase

*Acinetobacter parvus* HANDI 309 was originally isolated and identified from the feces of Korean Native calves, and the culture media was developed by Kim et al. [10]. The isolated culture was inoculated (50 mL) into culture medium (1 L) containing colloidal chitin agar medium with the following composition: 1% swollen chitin, 1% peptone and 1% NaCl (pH 5.0). Then, 1 mL of the spore suspension was added to 100 mL of colloidal broth, and incubated in a rotary incubator at 220 rpm and 30 °C for 120 h. After the incubation period, the culture broth was centrifuged at 10,000*g* and 4 °C for 20 min. The collected supernatant consisted of crude chitinase which was further used for analysis. The culture supernatant was used for chitinase assay after overnight dialysis against 10 μM sodium acetate buffer (pH 5.0). Cell growth was also measured at OD 660 nm.

### Enzyme activity assay by calorimetric method

For the determination of chitinase activity, colloidal chitin was used as a substrate. An amount of 0.5 mL of 1% colloidal chitin was added to 0.5 mL of enzyme solution and then the solution was incubated at 45 °C for 1 h. Next, 3 mL of 3,5-dinitrosalicyclic acid was added to stop the reaction, followed by incubation at 100 °C for 5 min. After centrifugation, the level of reducing sugar in the supernatant was carried out by the methods of Miller [11] with some modifications. The absorbance was noted at 540 nm using a UV spectrometer for the sample and also a blank. For the measurement of enzyme action, serial dilutions of GlcNAc was prepared and used. One millimole of GlcNAc was used as a standard.

### Chitinolytic activity by Petri dish method

The hydrolytic activity of the isolated HANDI 309 bacterial strain from the feces of calves was measured by the serial dilution method and was screened by its capability to produce hydrolytic enzymes using the plate method. The sterile culture medium was developed on nutrient agar plates supplemented with 0.1% colloidal chitin, and 1.5% agar medium was inoculated with the isolated organism at 30 °C for 24 h. After the incubation period, 0.1% Congo red solution was poured over the plate, and the clear zone around the isolates observed after the incubation period is an indication of chitinase enzyme production [12, 13].

### Molecular identification of *A. parvus* HANDI 309 for the production of chitinolytic enzyme

The 16S ribosomal DNA gene sequence was performed by method of Thompson et al. [14]. In brief, genomic DNA was isolated from *A. parvus* HANDI 309 and purified by a Wizard genomic DNA purification kit (Promega, USA). The subsequent genomic DNA amplifications were sequenced with Taq DNA polymerase using the universal primers of 27F (5′-AGA GTT TGA TCA TGG CTC AG-3′) and 1429R (5′-GGA TAT TAC GAC TTC TTG-3′). The amplified PCR products were purified by using the Wizard SV Gel and cleanup system (Promega). The purified PCR products were sequenced using an ABI PRISM 3730 DNA analyzer. The results were compared using sequence homology using the ClustalX program of Mega 2 and with the DNA sequence of ribosomal GENBANK using the BLAST program. During the similarity comparison, the sequence required an initial threshold of 99% homology when compared with the raw sequence.

### Analysis of the hydrolytic products of swollen chitin

#### Thin layer chromatography (TLC)

In a 150-μL reaction mixture containing 0.5 mL of 1% of the substrate and 0.5 mL of *A. parvus* HANDI 309 chitinases in 50 mM of phosphate buffer at pH 7.0, those reaction mixtures were incubated at 37 °C for 12 h. Next, 10 μL of the response mixture was transferred to an Eppendorf tube containing 10 μL of 0.1 N NaOH to stop the reaction, and samples were stored in −20 °C until further analysis. Then, 20-μL aliquots from the reaction mixture were added to the chromatograph on silica gel plates, with a solvent system containing *n*-propanol, methanol, and ammonia water [7:3:1 (v:v:v)], and the hydrolyzed products were analyzed by spraying the plate with aniline-diphenylamine reagent and baking at 180 °C using a hot air gun for 3 min.

#### High-performance liquid chromatography (HPLC)

Analysis of the hydrolytic products of swollen chitin by *A. parvus* HANDI 309 extracellular chitinases was carried out by incubating the recombinant enzymes with the swollen chitin. The reaction mixture was added to 50 mM sodium phosphate buffer at pH 7.0, and incubated at 40 °C and 1300 rpm for 12 h. After that, 75 μL of the response mixture was mixed with 75 μL of 70% acetonitrile to stop the reaction, and then the reaction mixture was centrifuged at 16,100*g* for 10 min at 4 °C to remove the undigested swollen chitin. The supernatant was further concentrated until the complete evaporation of the solvent without heating. The remains were dissolved in 20 μL of 35% acetonitrile and the reaction mixture was stored at −20 °C until further analysis. The reaction mixture was analyzed by isocratic HPLC at 25 °C using a Shimadzu 10ATvp UV/VIS HPLC system (Shimadzu, Tokyo, Japan) with a Shodex Asahipack NH2P-50 4E column (4.6 ID × 250 mm). A sample or 20 ml of the reaction mixture was injected into the HPLC using a Hamilton syringe (Hamilton, Bonadzu, Switzerland). The liquid phase consisted of 67% acetonitrile and 33% MilliQ water and the flow rate was set to 0.70 mL/min, with the eluted chitooligosaccharides monitored by reading the absorption at 210 nm.

### Production optimization of chitinase produced by *A. parvus* HANDI 309

#### Effect of incubation time and cell growth on chitinase activity

The effect of incubation time and cell growth on the activity of chitinase by *A. parvus* HANDI 309 was determined by using an inoculated flask containing the isolated culture inoculated in colloidal chitin agar medium, with the following composition (% w/v): colloidal chitin (1), yeast extract (0.1), K_2_HPO_4_ (0.07), KH_2_PO_4_ (0.03), MgSO_4_·7H_2_O (0.01), and FeSO_4_·7H_2_O (0.01) at pH 7.0, which was incubated using a rotary shaker at 200 rpm and 30 °C for 120 h. After that, the culture was centrifuged at 10,000 rpm for 20 min and the supernatant used for the chitinase activity. For the specific incubation time, the bacterial cells were grown for 120 h. The culture filtrate was harvested every 24 h and the enzyme production measured.

#### Impact of temperature and pH on chitinase activity

To determine the effect of incubation temperature and time, the incubating culture tryptic soy broth medium was measured at various temperatures (25, 30 and 35 °C) for 24 h on a shaking incubator (VS-8480SR; Vision Scientific, Korea) at 200 rpm. The effect of initial pH values on the chitinase production was analyzed by the inoculating broth culture medium composed of pancreatic digest of casein, 0.3% enzymatic digest of soy meal, 0.25% dextrose, 0.5% NaCl, and 0.25% dipotassium phosphate which was adjusted with 0.1 N HCl and 0.1 N NaOH to various initial pH of 5–9 for 24 h on a shaking incubator (VS-8480SR; Vision Scientific) at 200 rpm. Chitinase activity was measured as per the standard protocol, and the unit of chitinase activity was U/mL.

#### Effect of carbon source on chitinase activity

Four different types of carbon sources, glucose, sucrose, soluble starch and corn flour, were used to determine the impact of carbon sources on extracellular chitinase activity. These results were measured by adding different carbon sources (1% w/v) of supplemented media wihch were inoculated with inoculums (20 mL) fermented in optimized conditions. The culture medium contained different levels of glucose, which was adjusted to 0.5–2% on the shaking incubator. Chitinase activity was measured as per the standard protocol, and the unit of chitinase activity was U/mL. At the same time, the medium without carbon sources was used as a control.

#### Effect of organic and inorganic nitrogen source on chitinase activity

To explore the effect of organic and inorganic nitrogen sources on chitinase activity, various organic nitrogen sources (0.5% w/v), such as yeast extract, peptone, soybean flour and dry yeast were used, while the inorganic nitrogen sources (0.1% w/v) were urea, (NH_4_Cl_2_)SO_4_, NH_4_Cl, KNO_3_ and NaNO_3_, respectively. The different sources of both organic and inorganic sources inoculated with inoculums were incubated at pH 7.0 and 30 °C for 24 h under a shaking condition of 1200 rpm. The culture filtrate was used to measure chitinase activity after the incubation time, and the unit of chitinase activity was U/mole. An appropriate control was used for the study.

#### Effect of inorganic salts source on chitinase activity

The influence of various inorganic salts on chitinase activity was determined by inoculating the culture medium which was adjusted with different inorganic salts (1% w/v) of NaCl, K_2_HPO_4_, MgSO_4_·7H_2_O, MnSO_4_·5H_2_O and CaCl, and then incubated at pH 7.0 and 30 °C for 24 h under a shaking condition of 200 rpm. The culture filtrate was used to measure chitinase activity after the incubation time, and the chitinase activity was expressed as U/mol. An appropriate control was maintained for the above study.

## Results and discussion

In recent years, extracellular chitinases have received more attention due to their potential applications in industry. Therefore, many microbial extracellular chitinases have been isolated and characterized from various sources. However, there has been no previous report on the characterization of GlcNAc_6_ produced by a novel *A. parvus* HANDI 309 extracellular chitinase from the feces of calves. So far, about 10 morphologically different strains of chitinolytic enzyme-producing bacteria have been isolated from the feces of calves. Among them, *A. parvus* HANDI 309 exhibited the maximum degradation of swollen chitin and provided a clear zone. Therefore, *A. parvus* HANDI 309 was selected for further characterization of extracellular chitinase in the production of GlcNAc and the optimization of the production conditions. Hence, for the first time, we have described GlcNAc_6_ produced by a novel *A. parvus* HANDI 309 extracellular chitinase which was more active at 30 °C and 24 h at pH 7.0.

### Molecular identification of HANDI 309

The 16SrRNA sequence analysis of HANDI 309 was performed, and revealed the maximum sequence homology with *A. parvus* (phylogenic tree provided as Additional files 1, 2, 3, 4, 5). The acknowledged bacterium was Gram-negative, non-fermentative, non-motile and facultative aerobic nature. Numerous study reports on the industrial production of microbial chitinase enzymes are mostly based on the yield and applications. Therefore, this high-yielding extracellular chitinase producing a novel *A. parvus* HANDI 309 has been isolated, identified, genotypically confirmed and deposited in the Korean Agricultural Culture Collection (KACC91499P).

### Chitinolytic activity of extracellular chitinase enzyme

**Fig. 1:**
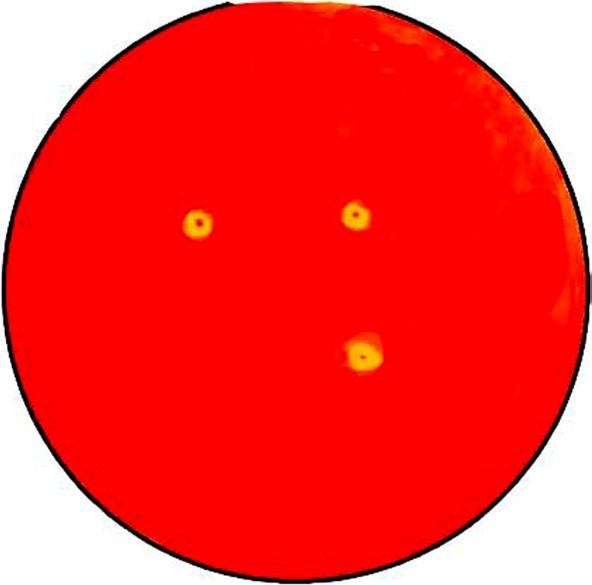
Screening of chitinolytic activity of isolated extracellular chitinase on agar medium containing chitin and stained with Congo red

The chitinolytic activity of *A. parvus* HANDI 309 extracellular chitinase was analyzed by using a Petri dish plate assay method. The chitinase hydrolyzed the 0.1% of swollen chitin in various concentrations after 24–72 h of growth on Congo red agar supplemented by swollen chitin as a carbon source. It produced a clear zone around the growing bacteria which indicated the chitinase activity (Fig. 1).

### Characterization of hydrolysis products by TLC and HPLC

**Fig. 2:**
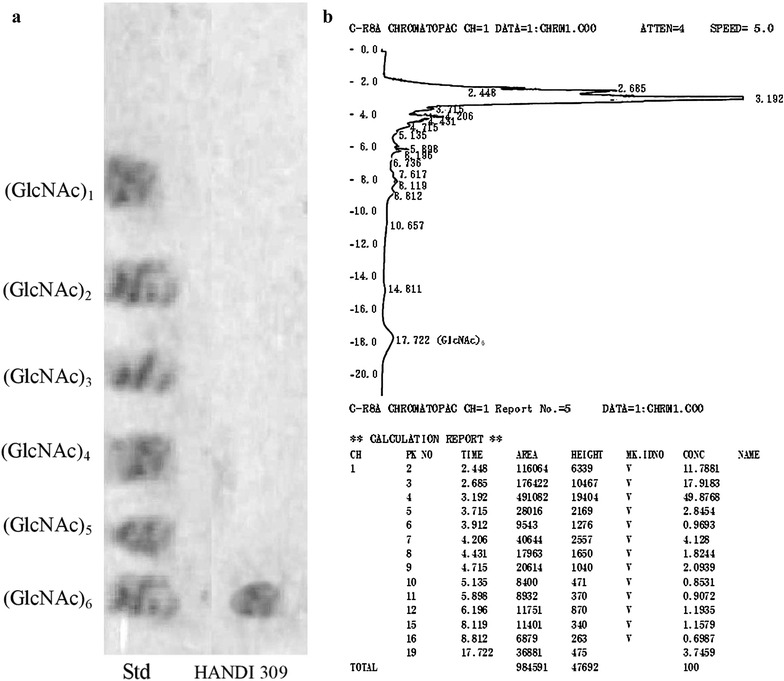
TLC (**a**) and HPLC profile (**b**) analysis of hydrolytic products of chitin oligosaccharides in the presence of *Acinetobacter parvus* HANDI 309 extracellular chitinase

The accumulated polysaccharides in chitin hydrolyzed by extracellular chitinase enzyme, and their end products of chitooligosaccharide, were identified by TLC as GlcNAc_6_ as shown in Fig. 2a. When the chitinolytic enzyme was incubated with swollen chitin, after 24-h experimental periods, GlcNAc_6_ was observed as the major end product. The eluted GlcNAc_6_ was further validated and quantified by using HPLC (Fig. 2b). Thus, *A. parvus* HANDI 309 extracellular chitinase is an ideal biocatalyst for microbial degradation and utilization of chitin in an industrial application which can more efficiently use it in bioconversion.

### Optimization of extracellular chitinase activity

#### Effect of chitin and cell growth on chitinase activity

**Fig. 3:**
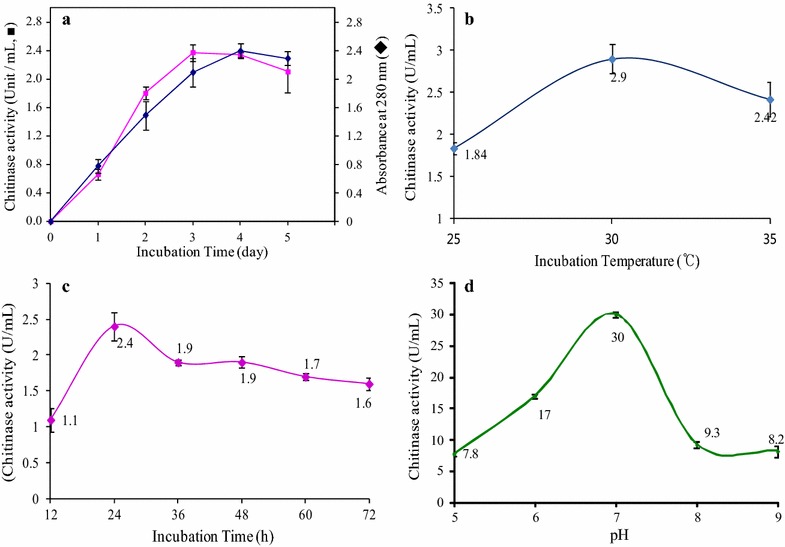
Effect of the cell growth (**a**) incubation temperature (**b**), time (**c**) and pH (**d**) of the culture medium on *A. parvus* HANDI 309-produced exo-chitinase activity

We observed that *A. parvus* HANDI 309 supports the maximum swollen chitin degradation by the production of extracellular chitinase enzyme in seed culture after 24 h incubation time (Fig. 3a). *Acinetobacter parvus* HANDI 309 produced extracellular chitinase showing the highest chitinolytic activity after 24 h (2.4 U/mL) which then remained constant up to 96 h after which we noted the activity of extracellular chitinase enzyme decreased. The reduced activity of chitinase enzyme is due to the inactivation of the secretary machinery of the enzyme, by the lack of nutrients or toxic agents present in the culture medium. This showed that the chitinase production is directly proportional to bacteria growth. From the study, we confirmed that the influence of swollen chitin concentration on the chitinase activity demonstrated that the chitin correlated with the chitin substrate concentration. Hence, the maximum activity was shown at 0.5% chitin concentration in the culture medium. Our results showed that a novel *A. parvus* HANDI 309 biofuel had a greater efficiency to produce exo-chitinase for the production of GlcNAc_6_ on an industrial scale as compared with the production of GlcNAc_6_ from chitin by *Aeromonas* sp. GJ-18-produced chitinase enzyme [15]. Similar results were shown by Nawani et al. [16], who reported that the chitinase activity correlated with the concentration of the swollen chitin. Also, the swollen chitin served as substrate or accumulation of chitin-decomposing intermediaries which served as a synthesis inhibitor of chitinases. The enzymatic reaction rate is directly proportional to the substrate concentration when at a low concentration. The greater concentration of the substrate has reduced the influence of the enzymatic reaction rate [17, 18].

#### Effect of temperature, time and pH on chitinase activity

In the bioprocess, temperature, time and pH are important environmental factors, which play a significant role in its regulation. Therefore, the bacteria growth and enzyme activity are also affected by changes in incubation temperature, time and pH. Hence, we determined the optimum temperature, time and pH for the growth of bacteria and chitinolytic enzyme activity. Figure 3b–d exhibits the *A. parvus* HANDI 309-produced exo-chitinase activity optimum temperature (30 °C), time (24 h) and pH (7.0), respectively. This result was supported by Frandberg and Schniirer [19], who showed that the maximum activity of *Bacillus pabuli K1* is obtained at 30 °C, whereas *Bacillus pabuli K1* did not produce any chitinases at 10 °C. Also, our results show that a novel *A. parvus* HANDI 309 biofuel had a greater efficiency to produce exo-chitinase as compared with the report of the production of GlcNAc_6_ from chitin by *Aeromonas* sp. GJ-18-produced chitinase enzyme [15]. From the above report, we have confirmed that a low temperature does not hamper the physiological functioning of the bacteria, including the enzymatic-concerning chitin. The present results concerning the time factor influencing chitinase activity point to the fact that *A. parvus* HANDI 309 underdetermination was able to decompose the maximum at 24 h. The chitinolytic bacteria used the numerous carbon and nitrogen sources, but on the other hand reater amounts of accumulated chitin may slow down the further production of chitinases. From the above study, we have confirmed that the chitin decomposable compound and microorganisms need a shorter time to adapt to the substrate and become able to start the production of chitinases. The pH of the medium not only plays a significant role in the manufacture of chitinase but also helps with the cell growth. Many studies have reported that the maximum yield of chitinases enzymes obtained from bacteria is at neutral or slightly acidic pH condition [20]. By optimizing the high culture, the status has increased the production of chitinase at the end of the stage. The present study revealed that the *A. parvus* HANDI 309 culture could improve the production of different metabolites by adopting these culture conditions.

#### Effect of carbon sources on chitinase activity

**Fig. 4:**
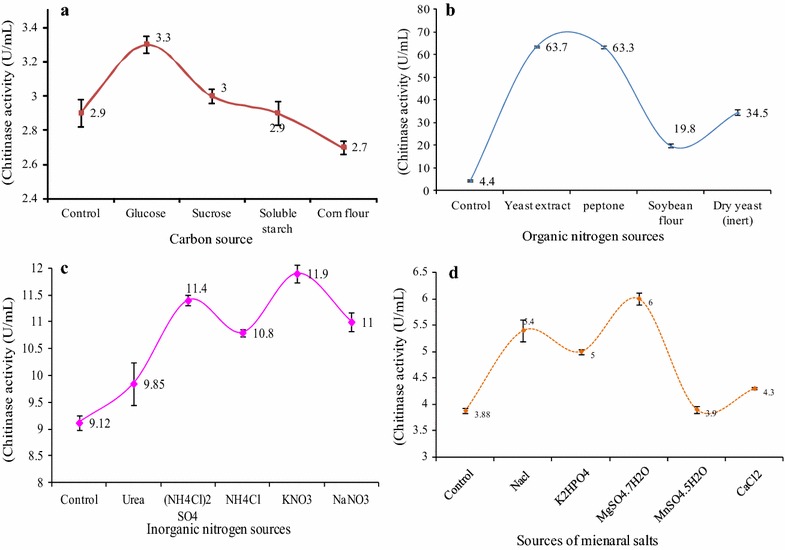
Effect of the carbon (**a**), organic (**b**), inorganic nitrogen (**c**) and mineral (**d**) sources of the culture medium on *A. parvus* HANDI 309-produced exo-chitinase activity

Carbon sources act as major constituents for the activity of extracellular chitinase. The bacteria growth medium supplemented with four different carbon sources (1%), glucose, sucrose, soluble starch and corn flour, were used to investigate the maximum chitinase production using swollen chitin as substrate. From the tested carbon sources, glucose supported the maximum extracellular chitinase activity from *A. parvus* HANDI 309 (3.3 U/mL) as compared with the other carbon sources of sucrose (3.0 U/mL), soluble starch (2.9 U/mL) and corn flour (2.7 U/mL), with the concentration of 0.5% of *A. parvus* HANDI 309 (Fig. 4a) enhancing the enzyme activity by acting as an inducer when also compared with sucrose, soluble starch and corn flour. The expression of chitinolytic chitinase is frequently induced by fungal cell wall components of glycoproteins and polysaccharides and reserved by carbon catabolic repressors of glucose and fructose [21]. The other supplements of swollen chitin such as chitin powder and chitin flakes are not an efficient source of swollen chitin, due to their colloidal nature. This present observation has been supported by Balakrishnan et al. [22] who obtained the maximum chitinase activity and protein content from *Streptomyces* sp. using 2% of sucrose.

#### Effect of organic and inorganic nitrogen sources on chitinase activity

Our results have shown the impact of various organic nitrogen sources on the activity of extracellular chitinase from *A. parvus* HANDI 309. Thus, the yeast extract exhibited the maximum chitinase activity of 63.7 U/mL when compared with the other nitrogen sources of peptone (63.3 U/mL), soybean flour (19.8 U/mL) and inert dry yeast (34.5 U/mL) with *A. parvus* HANDI 309 in the culture medium, and was improved with organic nitrogen sources such as 1% yeast extract (Fig. 4b) adjusted with concentrations of 0.05–2%. This study supports the results of Tagawa and Okazaki [23] that yeast extract was a favourable supplement of the fermentation medium for the manufacture of polysaccharides, and of Suresh and Chandrasekaran [24] who also reported that yeast extract enhances the yield of enzyme production with the supplementation of phosphate. This finding significantly supports that by Priya et al. [25] who used various organic nitrogen sources like ammonium, sulfate, peptone, yeast extract, swollen chitin and malt extract, with swollen chitin exhibiting the maximum chitinase activity. Also, to conclude the effect of various inorganic nitrogen sources, such as urea, (NH_4_Cl)_2_SO_4_, NH_4_Cl, KNO_3_ and NaNO_3_ used for the production of chitinase with *A. parvus* HANDI 309, KNO_3_ was the most favourable source and supported the maximum chitinase activity produced by *A. parvus* HANDI 309 (11.9 U/mL) as compared with urea (9.85 U/mL), (NH_4_Cl)_2_SO_4_ (11.4 U/mL), NH_4_Cl (10.8 U/mL) and NaNO_3_, with the adjusted concentration of KNO_3_ [0.1% (w/v)] (Fig. 4c). A greater activity of chitinase was developed by organic nitrogen compared with inorganic nitrogen. This is may be due to the presence of more amino acids and growth factors for bacteria growth which can be metabolized directly by cells. Hence, potassium nitrate can be an excellent alternative for the large-scale production of chitinase in industry.

#### Effect of inorganic salts on chitinase activity

Inorganic salts play a significant role in maintaining the cell growth, structure, configuration and activity of extracellular chitinase enzymes [26]. The activity of *A. parvus* HANDI 309 extracellular chitinase can be influenced by the presence of inorganic salt sources in the culture medium. Therefore, the effect of various inorganic salts on chitinase activity by *A. parvus* HANDI 309 was analyzed. The study result demonstrated the optimized concentration of inorganic salt which enhanced (Fig. 4d) the chitinase activity by incorporating the inorganic salt of MgSO_4_·7H_2_O at 0.05% (w/v) concentration as compared to other inorganic salts of NaCl (5.4 U/mL), K_2_HPO_4_ (5.0 U/mL), MgSO_4_5H_2_O (3.9 U/mL) and CaCl_2_ (4.3 U/mL). The enhancement of chitinase activity can be due to the presence of factors involving salts and enzyme production, which can ultimately lead to the chitinase activity. This study agreed with an earlier report wherein the results exhibited the agonist activity of MgSO_4_·7H_2_O on the manufacture of chitinase enzyme by *Pantoea dispersa* and *Streptomycetes* [3, 27]. The present study has demonstrated that the extracellular chitinase from *A. parvus* HANDI 309 could be capable of industrial applications.

## Conclusion

Extracellular chitinases play a crucial role in the pharmaceutical and biotechnological industries and especially in the production of fermented products. In conclusion, a novel bacterial strain of *A. parvus* HANDI 309 exo-chitinase can produce the *N*-acetyl-d-glucosamine (GlcNAc)_6_ at an large-scale in short time. The isolated *A. parvus* HANDI 309 exo-chitinases have the potential to hydrolyze the chitin efficiently, and it has the capability to reach maximum production of GlcNAc_6_ in 24 h with the optimal temperature 30 °C and pH 7.0 with carbon source glucose 0.5%, yeast extract 1.0%, inorganic nitrogen KNO_3_ 0.05%, and inorganic salt MgSO_4_ 0.06%. Therefore, these unique enzymatic properties of *A. parvus* HANDI 309 exo-chitinases can lead to the development of potentially low-cost alternatives in the pharmaceutical, food and biotechnological industries. In particular, further studies are needed to establish the low-cost, eco-friendly and effective biological method for the production of bioethanol from chitin by *A. parvus* HANDI 309 on an industrial scale.

## Additional files

**Additional file 1: Figure S1.** Phylogenetic analysis based on 16S rRNA gene sequences available from the National center for biotechnological information data library constructed after multiple alignments of data by ClustalX.

**Additional file 2: Figure S2.** Effect of glucose of the culture medium on the extracellular chitinase activity.

**Additional file 3: Figure S3.** Effect of yeast extract of the culture medium on the extracellular chitinase activity.

**Additional file 4: Figure S4.** Effect of KNO_3_ of the culture medium on the extracellular chitinase activity.

**Additional file 5: Figure S5.** Effect of magnesium sulfate of the culture medium on the extracellular chitinase activity.

## Abbreviations

CaCl: Calcium chloride
FeSO_4_·7H_2_O: Ferrous sulfate heptahydrate
GlcNAc: *N*-acetyl-β-d-glucosamine
HPLC: High-pressure liquid chromatography
K_2_HPO_4_: Dipotassium hydrogen phosphate
KACC: Korean agricultural culture collection
KH_2_PO_4_: Potassium dihydrogen phosphate
MgSO_4_·7H_2_O: Magnesium sulfate heptahydrate
NaNO_3_: Sodium nitrate
NaOH: Sodium hydroxide
TLC: Thin layer chromatography
