# Supplementary figures and images for: Novel Acinetobacter parvus HANDI 309 microbial biomass for the production of N-acetyl-β-d-glucosamine (GlcNAc) using swollen chitin substrate in submerged fermentation
Source: Biotechnol Biofuels. 2017 Mar 9;10:59. doi: 10.1186/s13068-017-0740-1 (PMC5345198; doi:10.1186/s13068-017-0740-1)

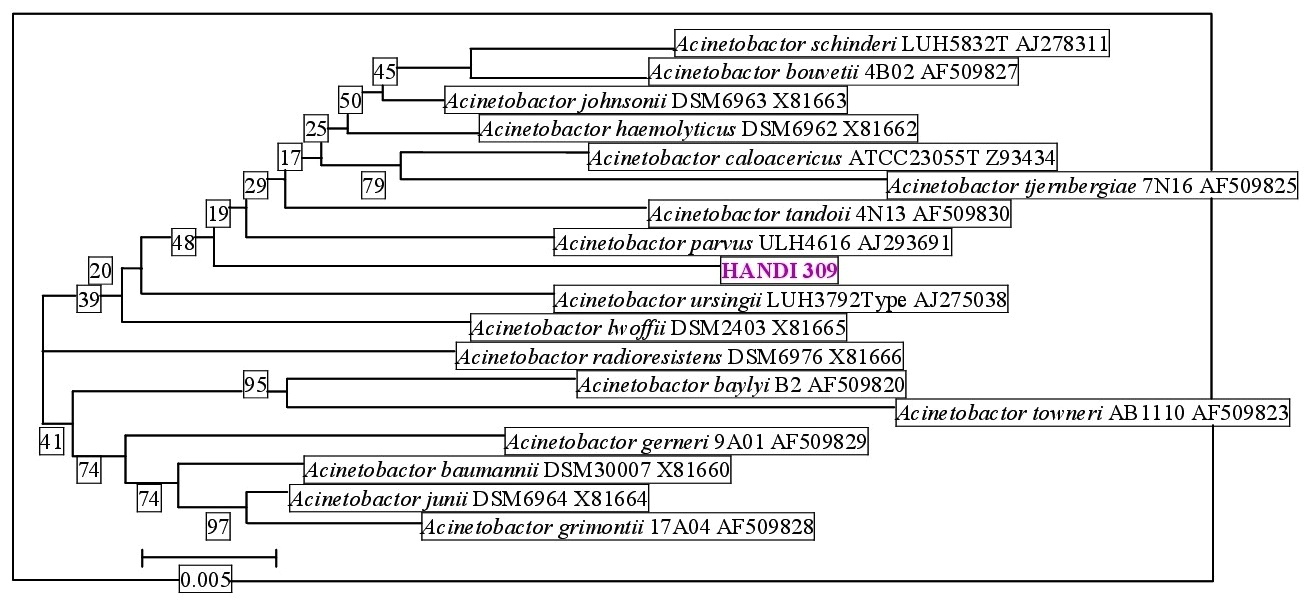

Supplement: Supplementary file 1 — Additional file 1: Figure S1. Phylogenetic analysis based on 16S rRNA gene sequences available from the National center for biotechnological information data library constructed after multiple alignments of data by ClustalX. [file 13068_2017_740_MOESM1_ESM.jpg]
